# Targeting RORα in macrophages to boost diabetic bone regeneration

**DOI:** 10.1111/cpr.13474

**Published:** 2023-04-13

**Authors:** Yufeng Shen, Qingming Tang, Jiajia Wang, Zheng Zhou, Ying Yin, Yifan Zhang, Wenhao Zheng, Xinyuan Wang, Guangjin Chen, Jiwei Sun, Lili Chen

**Affiliations:** ^1^ Department of Stomatology Union Hospital, Tongji Medical College, Huazhong University of Science and Technology Wuhan 430022 China; ^2^ School of Stomatology Tongji Medical College, Huazhong University of Science and Technology Wuhan 430030 China; ^3^ Hubei Province Key Laboratory of Oral and Maxillofacial Development and Regeneration Wuhan 430022 China; ^4^ Department of Stomatology, The First Affiliated Hospital, School of Medicine Shihezi University Shihezi 832000 China

## Abstract

Diabetes mellitus (DM) has become a serious threat to human health. Bone regeneration deficiency and nonunion caused by DM is perceived as a worldwide epidemic, with a very high socioeconomic impact on public health. Here, we find that targeted activation of retinoic acid‐related orphan receptor α (RORα) by SR1078 in the early stage of bone defect repair can significantly promote in situ bone regeneration of DM rats. Bone regeneration relies on the activation of macrophage RORα in the early bone repair, but RORα of DM rats fails to upregulation as hyperglycemic inflammatory microenvironment induced IGF1‐AMPK signalling deficiency. Mechanistic investigations suggest that RORα is vital for macrophage‐induced migration and proliferation of bone mesenchymal stem cells (BMSCs) via a CCL3/IL‐6 depending manner. In summary, our study identifies RORα expressed in macrophages during the early stage of bone defect repair is crucial for in situ bone regeneration, and offers a novel strategy for bone regeneration therapy and fracture repair in DM patients.

## Graphical abstract

Bone regeneration relies on the activation of macrophage RORα in the early bone repair. In the state of diabetes mellitus, RORα fails to upregulate as hyperglycemic inflammatory microenvironment induced IGF1‐AMPK signalling deficiency, leading to impaired bone repair. Activation of RORα by SR1078 in the early stage of bone defect repair can significantly promote the migration and proliferation of BMSCs via a CCL3/IL‐6 depending manner, thus boosting diabetic bone regeneration.

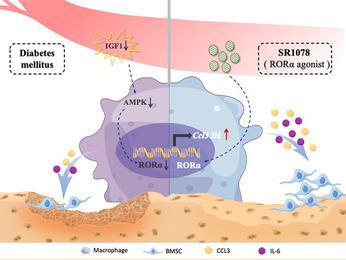

## 1 INTRODUCTION

Diabetes mellitus (DM) is one of the most common chronic metabolic diseases and the global prevalence of DM in 2019 is estimated to be 9.3% (463 million people), rising to 10.2% (578 million) by 2030 worldwide.^1^ The disruption of skeletal system is documented as the one of most common complications of DM.^2^, ^3^ DM patients often suffer from an explosive increase of bone fracture, and excessive hyperglycemia delays bone healing and high risk of bone graft implantation failure.^4^, ^5^, ^6^ Underlying mechanisms involved in diabetic bone regeneration deficiency are gradually recognized, for example dysfunction of immune cells (e.g., macrophages) and impaired osteoblast function, etc.^6^ Accordingly, some therapeutic intervention approaches to facilitate bone healing under diabetic conditions have been proposed, including local application of anti‐inflammatory cytokines, hyperbaric oxygen therapy or implantation of isogenic adult stem cells (ASCs).^7^, ^8^, ^9^ Unfortunately, bone regeneration of DM remains a clinical challenge, with defect of stem cells in a high‐glucose microenvironment being the primary obstacle.^10^ Hence, it is imperative to develop an effective strategy to recruit autologous stem cells to improve osteogenesis in DM patients.

Persistent non‐resolving inflammation, characterized by explosive increase of leukocytes (e.g., macrophages) and proinflammatory cytokines, is the main underlying mechanism to defect of stem cells and impaired healing of DM.^11^, ^12^ Paradoxically, trauma microenvironment of DM is completely different during the acute phase of healing in that it suffers from inadequate macrophages and insufficient inflammatory response.^13^, ^14^, ^15^, ^16^ What causes macrophages hypofunction and stem cell deficiency in diabetic bone defect during the acute phase of healing remains unknown, prompting us to revisit this issue? Retinoic acid‐related orphan receptor α (RORα) is a multi‐faceted nuclear receptor in tissue regeneration beyond an ability to regulate immune signalling.^17^ A significant body of work has focused on the roles of the RORs, and elegant genetic studies have established that RORα expression is closely related to DM and is indispensable to orchestrate immune microenvironment and osteogenesis.^18^, ^19^, ^20^, ^21^ Therefore, we speculated that RORα may be a vital factor in regulating inflammatory microenvironment in the early stage of bone defect and inflammatory imbalance in DM, providing a novel target for treating diabetic bone regeneration deficiency.

In this study, we found that RORα expressed in macrophages is essential for in situ bone regeneration. Targeted activation of RORα by SR1078 in the early stage of bone defect boosts bone regeneration of DM rats. Macrophage RORα fails to upregulate as hyperglycemic inflammatory microenvironment induced insulin‐like growth factor 1 (IGF1) scarcity and 5′‐AMP‐activated protein kinase (AMPK) signalling inactivation in the early stage of bone defect repair from DM rats, which causes regeneration deficiency severely. RORα is vital for macrophage‐induced migration and proliferation of BMSCs via a C‐C motif chemokine 3 (CCL3)/interleukin‐6 (IL‐6) depending manner. Overall, our study thus provides newly fundamental insights into the osteogenesis under DM conditions and offers a novel strategy for bone regeneration therapy in diabetic patients.

## 2 RESULTS

### 2.1 Activation of RORα by SR1078 boosts in situ bone regeneration of DM rats

**FIGURE 1:**
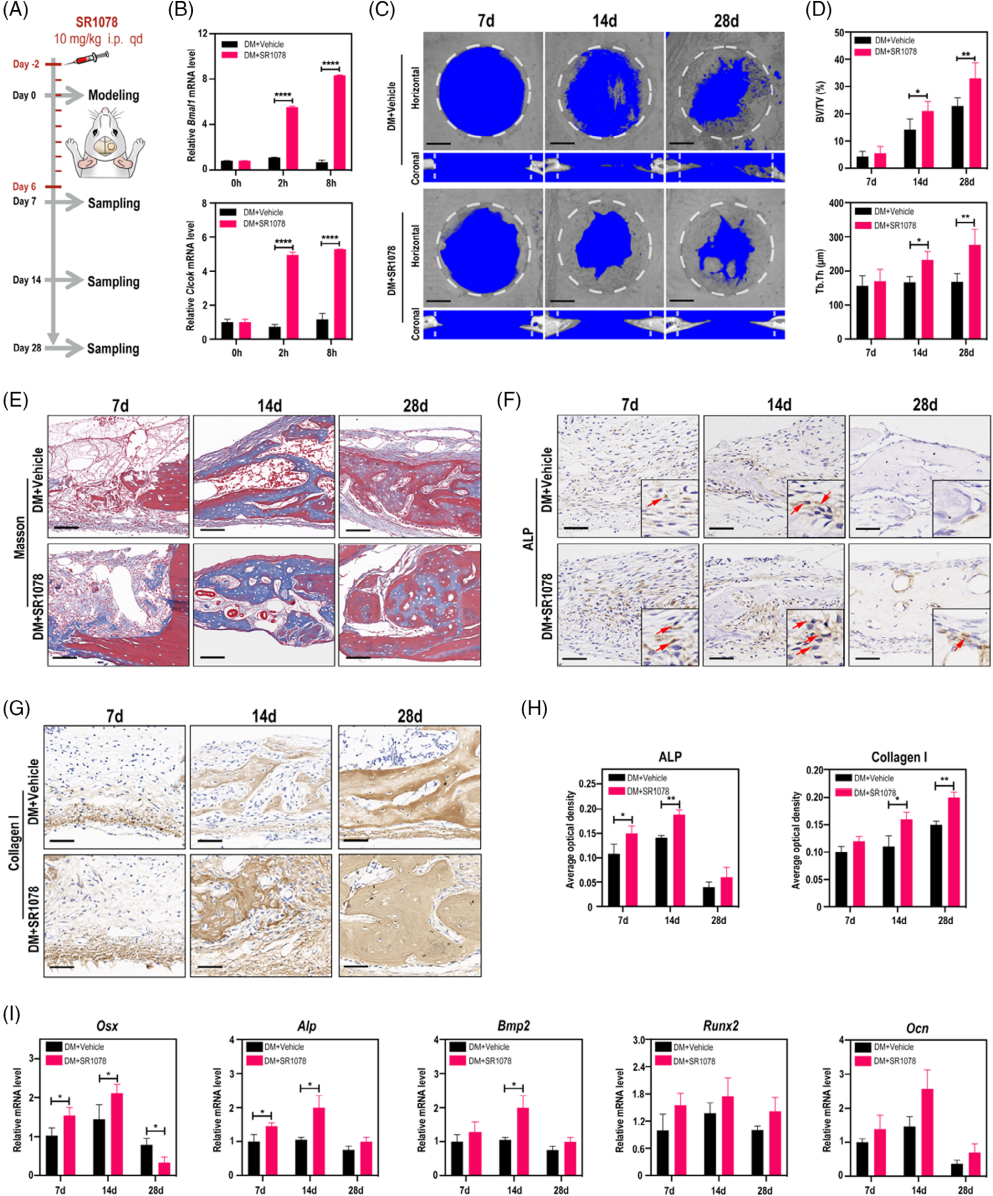
Activation of RORα by SR1078 Boosts in situ bone regeneration of DM rats. (A) Schematic diagram of the experiment. (B) qRT‐PCR analysis of *Bmal1 and Clock* mRNA in calvarial bone tissues of DM rats at 0, 2 and 8 h post SR1078 injection. (C) Micro‐CT scanning of calvarial defects on days 7, 14 and 28 post surgery. The 4 mm‐diameter defect area (white dashed lines) was selected as the region of interest (ROI). Scale bar =1 mm. (D) BV/TV and Tb.Th analysis of the selected ROI. (E) Masson staining of calvarial defects on days 7, 14 and 28 post surgery. Scale bar = 100 μm. (F‐H) IHC staining of ALP (F) and Collagen I (G) in calvarial defects and corresponsive quantity analysis (H). Scale bar = 50 μm. (I) qRT‐PCR analysis of *Osx*, *Alp*, *Bmp2*, *Runx2* and *Ocn* in calvarial bone tissues on days 7, 14 and 28 post surgery. *p < 0.05, **p < 0.01, ****p < 0.0001.

To test whether activation of RORα could promote DM bone regeneration, we established a calvarial defect model in type 2 DM rats and SR1078, a selective agonist of RORα, was administered to activate RORα driven transcription (Figure 1A). *Bmal1* and *Clock* are the main target genes of RORα, and qRT‐PCR assays firstly indicated that the mRNA transcript of *Bmal1* and *Clock* in the calvarial bone was increased 2‐h after SR1078 injection, and the increasement was more significant after 8‐h, suggesting that SR1078 was existing in the calvarial defect (Figure 1B). Micro‐CT analysis showed limited bone healing in the DM rats, with less than 30% new bone in the defect area after 28 days (Figure 1C,D). Significantly, the amount of new bone in the defect area at day 14 in the SR1078 group was comparable to that in the vehicle group at day 28, indicating an accelerated osseous regeneration by SR1078, which was evidenced by bone volume per tissue volume (BV/TV) and trabecular thickness (Tb.Th) measurement (Figure 1C,D). Masson staining showed that the newly formed bone marked by red was much more in the SR1078 group (Figure 1E). To further assess the osteogenesis at molecular biology, we conducted alkaline phosphatase (ALP) and type I collagen (COL1A1) IHC staining, which were the markers of early and late osteogenesis, respectively. The staining data showed remarkably higher osteogenesis activities in the SR1078 group during the whole healing period (Figure 1F–H). QRT‐PCR data showed that the mRNA levels of osteogenesis indicators *Osx*, *Alp*, Bone morphogenetic protein 2 (*Bmp2*), Runt‐related transcription factor 2 (*Runx2*) and Osteocalcin (*Ocn*) were obviously up‐regulated in the SR1078 group compared with the Vehicle group (Figure 1I). Taken together, these results suggested that functional activation of RORα by SR1078 can significantly promote in situ bone regeneration of DM rats.

### 2.2 RORα expressed in macrophages of DM rats is deficient in early bone repair

**FIGURE 2:**
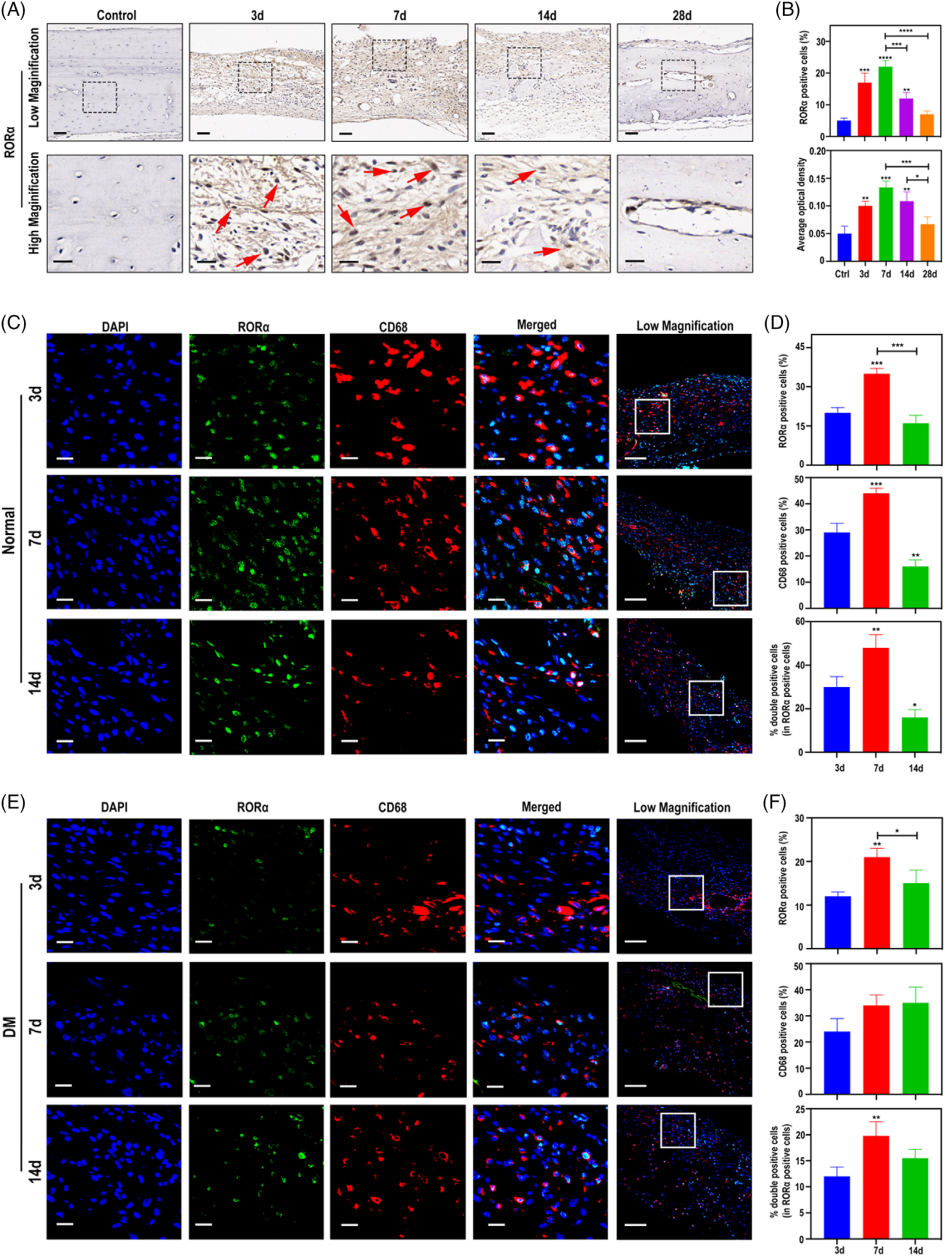
RORα expressed in macrophages of DM rats is deficient in early bone repair. (A) IHC staining of RORα in calvarial defects of normal rats on days 3, 7, 14 and 28 post surgery. Rats that received sham operation were taken as the control group. Red arrows show positive cells with intranuclear RORα staining. Low magnification scale bar = 50 μm and high magnification scale bar = 20 μm. (B) Quantification of RORα expression from the IHC images. (C, E) IF staining of RORα (green) and CD68 (red) in calvarial defects of normal rats (C) and DM rats (E) on days 3, 7, 14 post surgery. Scale bar =100 μm. (D, F) Quantitative analysis of RORα^+^, CD68^+^and RORα^+^CD68^+^ cells in the normal rats (D) and DM rats (F). *p < 0.05, **p < 0.01, ***p < 0.001, ****p < 0.0001.

To reveal the underlying pro‐regenerative effect of SR1078, we detected the expression change of RORα in the cranial defect tissue of normal rats and DM rats at 3, 7, 14 and 28 days post‐operatively. IHC staining showed low expression of RORα in the normal control rats and positive expression of RORα could be seen as early as 3 days after calvarial defect (Figure 2A,B). Marked increasement of RORα continued to day 7 and decreased afterwards (Figure 2A,B). As RORα‐positive cell morphology was biased towards macrophages, we surmised that RORα in calvarial tissue is mainly derived from macrophages. To test this, IF double staining for CD68, a pan macrophage marker, and RORα was carried out. We found that the overlap rate of the two fluorescence is high and CD68‐positive cells showed absolutely high RORα level in contrast to the stroma cells (Figure 2C,D). RORα staining intensity peaked at day 7‐post modelling in the normal group consistent with the IHC results and the percent of double positive cells within RORα‐positive cells showed the same tendency (Figure 2C,D). In DM rats, the proportion of CD68‐positive cells in the bone defect area was not significantly decreased compared to normal mice. However, RORα expressed in CD68‐positive cells in the DM group was lower than that in the normal group at all time points and lacked an early tendency to increase (Figure 2E,F), suggesting that RORα in macrophages is inhibited by DM microenvironment. Together, we speculated that RORα fails to increase physiologically in the early stage of bone defect repair from DM rats, which may be a vital cause of diabetic regeneration deficiency.

### 2.3 Inhibition of RORα by SR3335 impedes physiological in situ bone regeneration

**FIGURE 3:**
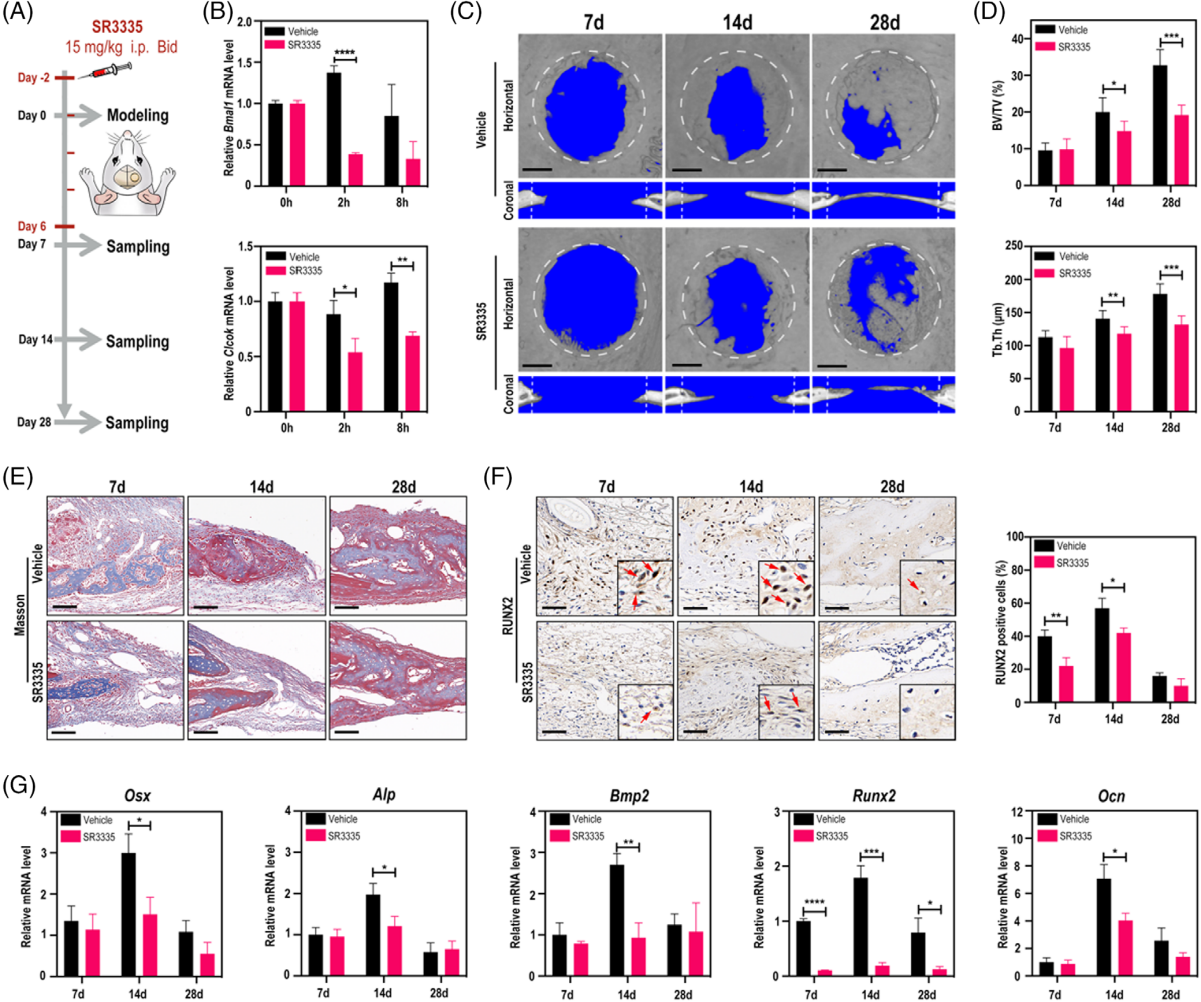
Inhibition of RORα by SR3335 impedes physiological in situ bone regeneration. (A) Schematic diagram of the experiment. (B) qRT‐PCR analysis of *Bmal1 and Clock* mRNA in calvarial bone tissues of normal rats at 0, 2 and 8 h post SR3335 injection. (C) Micro‐CT scanning of calvarial defects on days 7, 14 and 28 post surgery. The 4 mm‐diameter defect area (white dashed lines) was selected as the region of interest (ROI). Scale bar =1 mm. (D) BV/TV and Tb.Th analysis of the selected ROI. (E) Masson staining of calvarial defects on days 7, 14 and 28 post surgery. Scale bar = 100 μm. (F) IHC staining and analysis of RUNX2 in calvarial defects. Scale bar = 50 μm. (G) qRT‐PCR analysis of *Osx*, *Alp*, *Bmp2*, *Runx2* and *Ocn* in calvarial bone tissues on days 7, 14 and 28 post surgery. *p < 0.05, **p < 0.01, ***p < 0.001, ****p < 0.0001.

To further test the function of RORα in the physiological bone regeneration process, SR3335, an inverse agonist of RORα, was performed to suppress the constitutive transactivation activity of RORα during the early stage of bone healing in the normal rats (Figure 3A). qRT‐PCR results of *Bmal1* and *Clock* in the calvarial bone confirmed the efficacy of SR3335 (Figure 3B). Micro‐CT analysis showed that the amount of new bone in the defect area of the rats in the vehicle group increased significantly while no notable rise was observed in the SR3335 group from day 14 to 28, suggesting an impeded bone repairing process (Figure 3C,D). Masson staining indicated that the newly formed bone marked by red in the SR3335 group was less than that in the vehicle group at day 28 (Figure 3E). We speculated that the difference in the different groups may be due to the impact on osteogenesis of bone defects after intervention of RORα. To confirm this hypothesis, IHC staining of RUNX2 was carried out and the results showed that RUNX2 expression in the SR3335 group was lower than that in the vehicle group (Figure 3F). qRT‐PCR assays showed that osteogenesis markers *Osx*, *Alp*, *Bmp2*, *Runx2* and *Ocn* were remarkably down‐regulated after SR3335 administration (Figure 3G), suggesting attenuated osteoblast function after pharmacological inhibition of RORα. To be summarized, these results indicated that RORα is an essential player for physiological in situ bone regeneration.

### 2.4 Insufficient IGF1‐AMPK signalling of DM rats blocks upregulation of RORα

**FIGURE 4:**
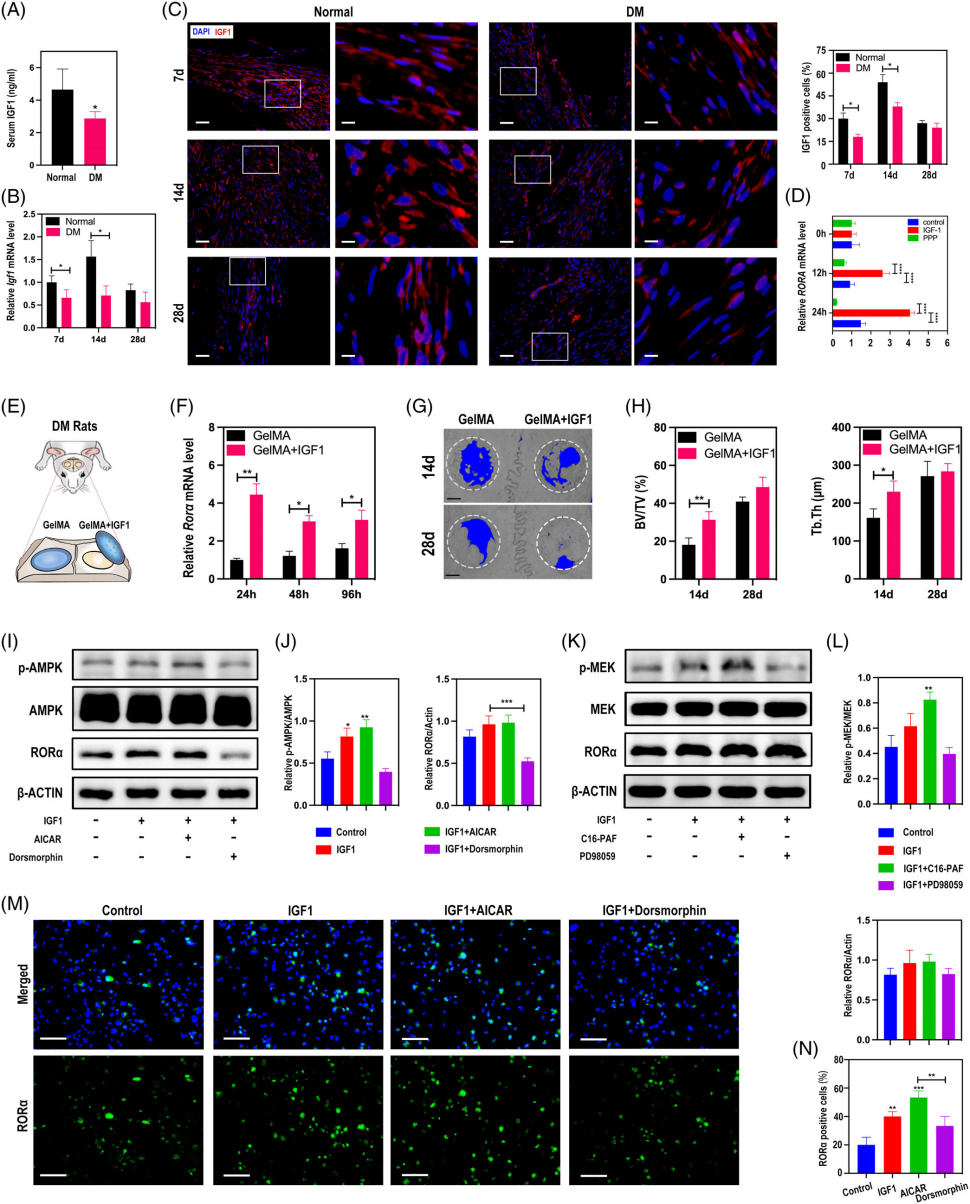
Insufficient IGF1‐AMPK signalling of DM rats blocks upregulation of RORα. (A) IGF1 content in the serum of normal and DM rats was detected by ELISA. (B) qRT‐PCR analysis of *Igf1 m*RNA levels in calvarial tissues on days 7, 4 and 28 post surgery. (C) IF staining and quantitative analysis of IGF1 in calvarial defects from normal and DM rats on days 7, 14, and 28 post surgery. Low magnification scale bar = 100 μm and high magnification scale bar = 25 μm. (D) THP‐1 derived macrophages cultured in 25 mM glucose containing medium were treated with IGF1 (100 ng/mL) or IGF1R inhibitor PPP (5 μM) for 12, 24 h and *RORA* mRNA levels were examined by qRT‐PCR. (E) Schematic illustration of topical administration of IGF1 in calvarial defects of DM rats. (F) qRT‐PCR analysis of *Rorα* mRNA in calvarial bone tissues at 24, 48, 96 h post IGF1 administration. (G) Micro‐CT scanning of calvarial defects on days 14, 28 post IGF1 administration. The 4 mm‐diameter defect area (white dashed lines) was selected as the region of interest (ROI). Scale bar =1 mm. (H) BV/TV and Tb.Th analysis of the selected ROI. (I) THP‐1 derived macrophages cultured in 25 mM glucose containing medium were pretreated with IGF1 (100 ng/mL) for 1 h followed by administration of AMPK activator (AICAR, 0.5 mM) or AMPK inhibitor (Dorsomorphin, 2.0 μM) for 24 h. The relative protein levels of p‐AMPKα1, AMPKα1 and RORα were detected by Western Blot. (J) Quantitative analysis of AMPKα1 phosphorylation and RORα levels. (K) THP‐1 derived macrophages cultured in 25 mM glucose containing medium were pretreated with IGF1 (100 ng/mL) for 1 h followed by administration of MAPK activator (C16‐PAF, 1.0 μM) or MAPK inhibitor (PD98059,10.0 μM) for 24 h. The relative protein levels of p‐MEK, MEK and RORα were detected by Western Blot. (L) Quantitative analysis of MEK phosphorylation and RORα levels. (M) IF staining of RORα in THP‐1 derived macrophages with different treatments and quantitative analysis (N). *p < 0.05, **p < 0.01, ***p < 0.001, ****p < 0.0001.

Deficiency of insulin‐like growth factor 1 (IGF1) is one hallmarker of the diabetic microenvironment, and its expression is sharply upregulated in early bone repair of normal individuals.^22^, ^23^ Hereby, we supposed that inhibition of RORα in DM may be due to IGF1 abnormity. We first detected the level of IGF1 in the serum of normal and DM rats by ELISA, and the results showed that IGF1 was significantly reduced in the serum of DM rats (Figure 4A). Further, we investigated the expression of IGF1 in calvarial defect region in rats. qRT‐PCR analysis illustrated that IGF1 expression was significantly lower throughout the whole bone healing process in the DM group compared with the normal group (Figure 4B). The most significant difference was observed at day 14, with a nearly 50% decrease (Figure 4B). IF staining results showed the change more visually (Figure 4C). We next explored whether IGF1 could regulate RORα in macrophages. THP‐1, a human monocyte‐derived cell line, were treated with IGF1 or IGF1 combined with IGF1R inhibitor PPP for 12, 24 h and mRNA level of *RORA* was detected. qRT‐PCR data indicated that IGF1 remarkably upregulated *RORA* transcription, which could be eliminated by PPP administration, suggesting a positive regulation role of IGF1 on RORα (Figure 4D). Moreover, we explored the regulation of IGF1 on RORα in vivo. Diabetic rats received calvarial surgery and IGF1 loaded in methylpropenyl acylated gelatin (GelMA) was applied topically (Figure 4E). We could clearly see that *Rorα* expression increased significantly by IGF1 in newly formed tissue (Figure 4F). Consistently, Micro‐CT analysis showed that more newly formed bone can be seen in the GelMA+IGF1 group at day 14 and day 28, compared with the GelMA group (Figure 4G,H). These findings suggested that IGF1 is the vital activator of RORα in early bone repair. It is well known that adenosine monophosphate‐activated protein kinase (AMPK) and mitogen‐activated protein kinase (MAPK) pathways are the classical downstream intracellular signal pathways of IGF1,^24^, ^25^ so we tested whether IGF1 regulated the expression of RORα by these two pathways. AMPK activator AICAR promoted phosphorylation of AMPK and RORα expression in THP‐1 cells (Figure 4I,J). Administration of AMPK inhibitor Dorsomorphin after IGF1 restrained the upward trend of AMPK phosphorylation and markedly inhibited RORα expression (Figure 4I,J). Similarly, MAPK activator C16‐PAF and inhibitor PD98059 were applied to examine the effect of MAPK on RORα. However, no significant difference was observed in the expression of RORα either by activation or inhibition of MAPK signalling (Figure 4K,L), suggesting that the regulation of IGF1 on RORα was independent of MAPK pathway. Moreover, IF staining reconfirmed the IGF1‐AMPK‐RORα axis (Figure 4M,N). These results indicated that IGF1 may regulate the expression of RORα through AMPK rather than MAPK.

### 2.5 RORα actuates macrophages‐induced migration and proliferation of BMSCs

**FIGURE 5:**
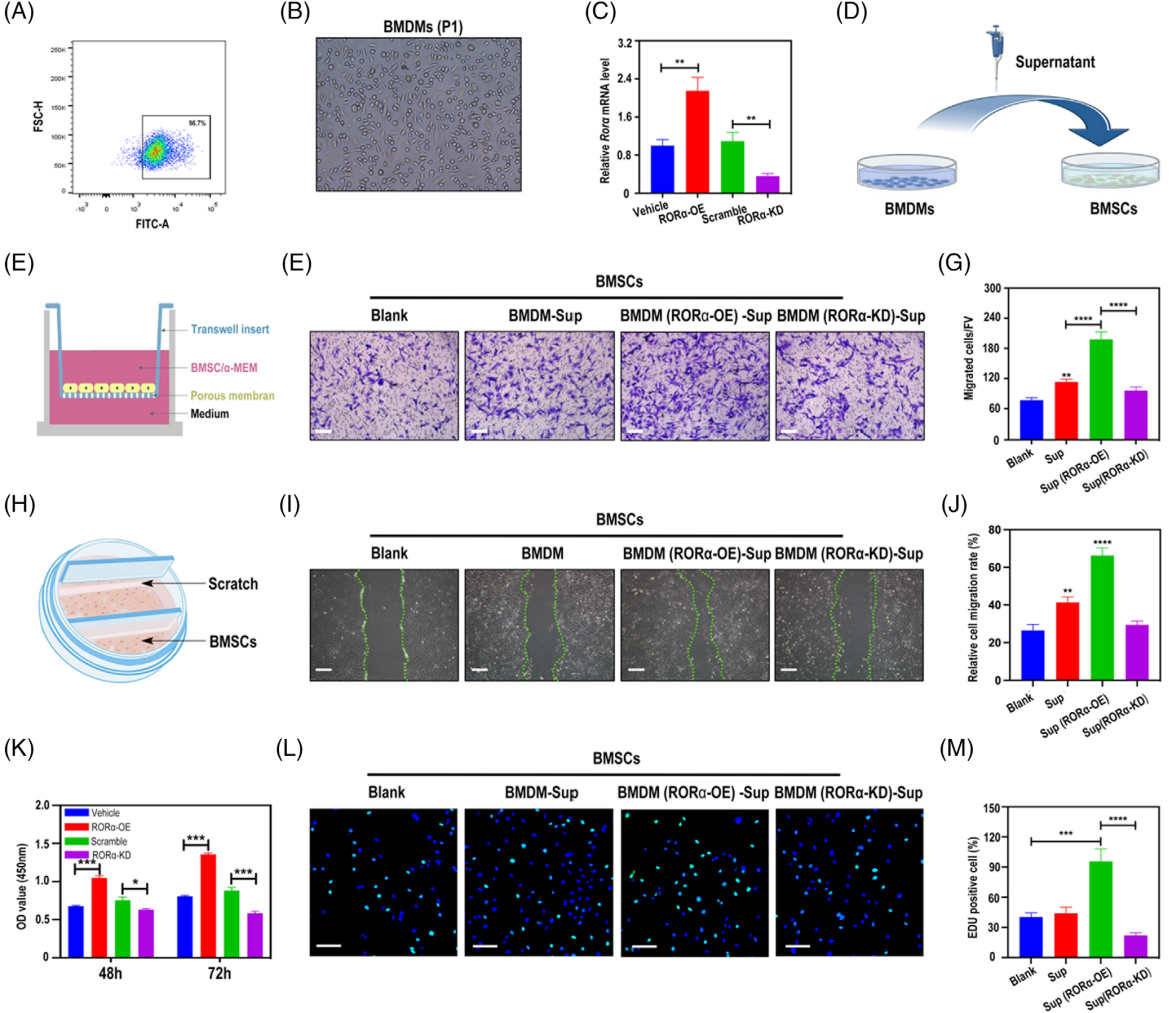
RORα actuates macrophages‐induced migration and proliferation of BMSCs. (A) Flow cytometry was used to identify the primary cultured rat BMDMs with anti‐CD68. (B) Representative images of rat BMDMs in P1 generation under light microscopy. (C) *Rorα* in BMDMs was over‐expressed via lentivirus or knocked down via CRIPER/Cas 9 system and the efficiencies were examined by qRT‐PCR. (D) Operation diagram of the co‐culture system. *Rorα*‐overexpressed or knockdowned BMDMs were culture for 48 h and the supernatant was saved as conditioned medium to culture BMSCs. (E) Schematic diagram of the transwell system. (F) BMSCs were incubated in conditioned medium from *Rorα*‐overexpressed or knockdowned BMDMs and the vertical migrated BMSCs were stained with crystal violet. Scale bar = 200 μm. (G) Quantitative analysis of transwell assay. (H) Schematic diagram of the scratch assay. (I) Horizontal migration of BMSCs in different conditioned media was determined by scratch assay. Scratch borders were indicated by green dashed lines. Scale bar =500 μm. (J) Quantitative analysis of scratch assay. (K) BMSCs were cultured in different conditioned media for 48, 72 h and the rates of cell growth were examined by CCK8 assay. (L) EDU staining of BMSCs cultured in different conditioned media and quantitative analysis (M). Scale bar =100 μm. *p < 0.05, **p < 0.01, ***p < 0.001, ****p < 0.0001.

After the appearance of bone defect, macrophages can rapidly recruit BMSCs through secreting chemokines, and BMSCs undergo osteogenic differentiation and exert bone regeneration effects.^6^ Therefore, we tested whether RORα is involved in the regulation of macrophages on BMSCs. Primary bone marrow derived macrophages (BMDMs) were isolated from SD rats and identified by flow cytometry of CD68 (Figure 5A,B). We overexpressed or knocked down *Rorα* in BMDMs, respectively, and the efficiencies were verified by QRT‐PCR (Figure 5C). Cellular supernatant of *Rorα*‐overexpressing or *Rorα*‐knockdown BMDMs was used as conditioned medium to incubate BMSCs (Figure 5D). Using a transwell co‐culture model (Figure 5E), we found that BMDMs‐conditioned medium promoted vertical migration of BMSCs (Figure 5F,G). This migration‐promoting effect was dramatically enhanced by RORα overexpression and abolished by RORα knockdown (Figure 5F, G). Scratch assay was also performed in specially designed 6‐well plates (Figure 5H). Similar with the results of transwell test, images and quantitative analysis of scratch assay showed that overexpression of RORα strengthened BMDMs‐mediated BMSCs horizontal migration whereas knockdown of RORα inhibited this process (Figure 5I,J). We also investigated the effect of RORα in BMDMs on BMSCs proliferation. CCK8 test demonstrated that after 48‐h or 72‐h incubation, the proliferation capacity of BMSCs treated with *Rorα*‐overexpressing conditioned medium was remarkably upregulated, while *Rorα*‐knockdown conditioned medium impaired BMSCs proliferation (Figure 5K). This result was further intuitively confirmed by EDU assays (Figure 5L,M). In summary, these results showed that RORα is vital for BMDMs to induce migration and proliferation of BMSCs.

### 2.6 CCL3/IL‐6 secreted by BMDMs transfer the RORα signalling to BMSCs

**FIGURE 6:**
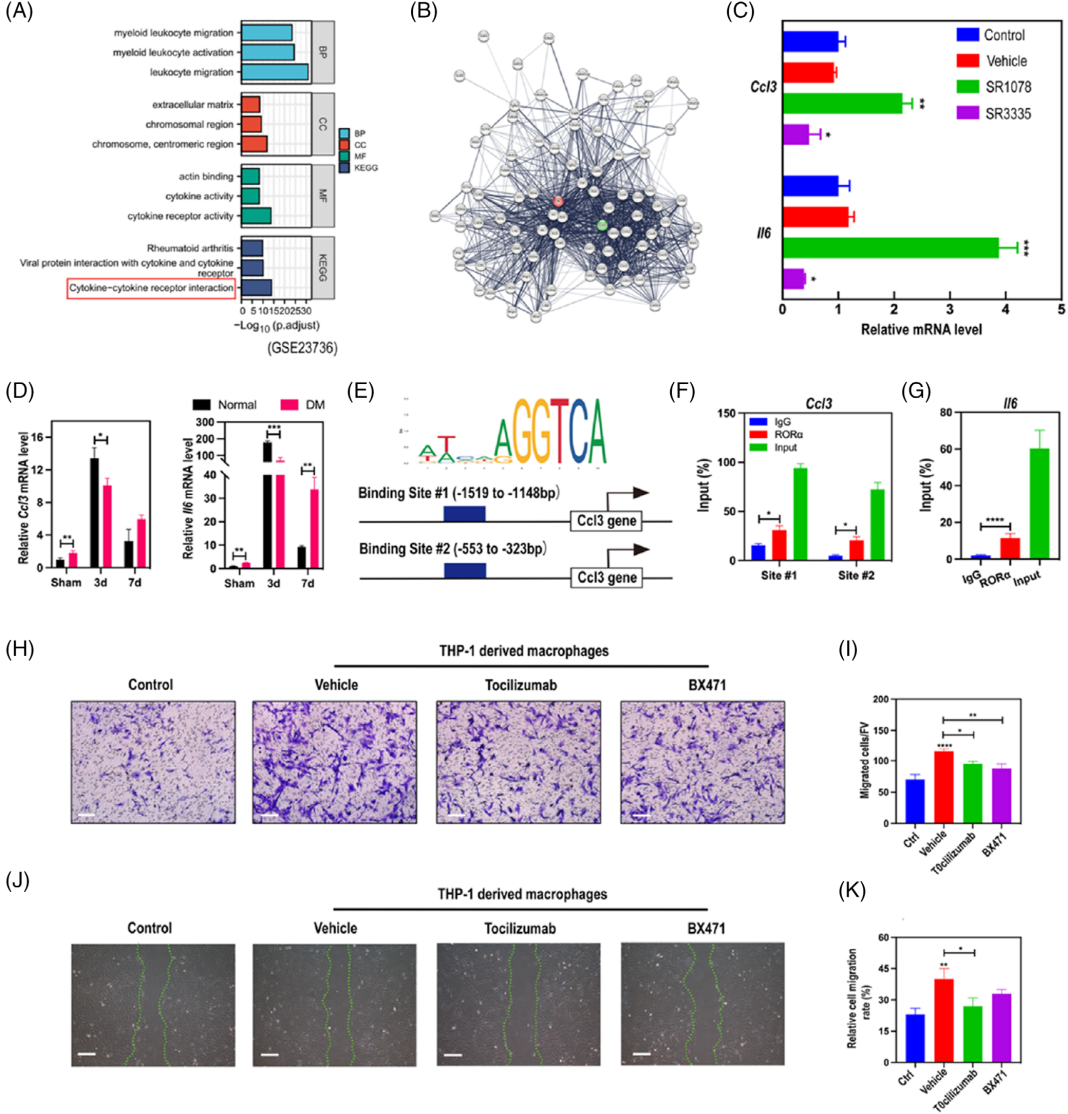
CCL3/IL‐6 secreted by macrophages transfer the RORα signalling to BMSCs. (A) GO and KEGG pathway enrichment analysis of the gene expression profiles from GSE23736 dataset. (B) PPI network of the DEGs in ‘Cytokine‐cytokine receptor interaction’ pathway. (C) RORA in THP‐1 derived macrophages was activated by SR1078 (5 μM) or inhibited by SR3335 (2 μM) and *Ccl3*, *Il6* mRNA level were examined by qRT‐PCR. (D) qRT‐PCR analysis of *Ccl3* and *Il6* mRNA level in calvarial defects of normal and DM rats on days 3, 7 post surgery. (E) Schematic diagram of the potential binding site for RORα in the promoter region of CCL3 using JASPAR database. (F, G) ChIP‐qPCR assay of CCL3 (F) and IL‐6 (G). (H) Conditioned medium was collected from THP‐1 derived macrophages treated with SR1078. Transwell assay was used to evaluate invasion ability of BMSCs incubated in conditioned medium supplemented with CCL3‐neutralizing antibody (BX471, 1 μM) or IL6‐neutralizing antibody (Tocilizumab, 1 μM) for 24 h. Scale bar = 200 μm. (I) Quantitative analysis of the transwell assay. (J) Scratch assay of BMSCs cultured in conditioned medium with or without neutralizing antibodies of CCL3/IL‐6 and quantitative analysis (K). Scale bar = 500 μm. *p < 0.05, **p < 0.01, ***p < 0.001, ****p < 0.0001.

To investigate the mechanism underlying the RORα‐induced BMSCs recruitment, we searched and obtained gene expression data for wild type (WT) and *Rorα*‐deficient mice fed with a high fat diet (GSE23736). After identifying differentially expressed genes (DEGs), we performed gene ontology (GO) and Kyoto Encyclopedia of Genes and Genome (KEGG) pathway enrichment to confirm the function of DEGs. The KEGG pathway ‘Cytokine‐cytokine receptor interaction’ was significantly down‐regulated in RORα‐deficient mice (Figure 6A). We then constructed protein–protein interaction (PPI) network to display the DEGs of “Cytokine‐cytokine receptor interaction” pathway and found *Ccl3* and *Il‐6* were among the most highly connected genes (Figure 6B). Based on this result, we speculated that *Ccl3* and *Il‐6* may be underlying target genes that are responsible for the biological function of RORα in BMDMs. qRT‐PCR analysis indicated that SR1078 remarkably increased *Ccl3* and *Il‐6* mRNA transcription in THP‐1 cells while SR3335 downregulated transcription of these two genes (Figure 6C), suggesting positive transcription regulation of RORα on *Ccl3* and *Il‐6*. In the DM calvarial defect model, *Ccl3* and *Il‐6* mRNA levels were lower than those in normal individuals in early bone repair, which was in line with RORα expression (Figure 6D). Then, we performed JASPAR analysis, identified RORE sites of RORα (Figure 6E) and predicted possible RORα binding sites in the promoter region of *Ccl3* (Figure 6F). Further CHIP‐qPCR assays confirmed RORα‐binding sites on *Ccl3* (Figure 6F). The transcriptional regulation of *Il‐6* by RORα was explored in a previous report^26^ and we verified the binding by CHIP‐qPCR analysis (Figure 6G). These results suggested that RORα may alter the transcriptional activity of *Ccl3* and *Il‐6* by direct binding. Next, we tested whether CCL3 and IL‐6 are essential for RORα‐mediated recruitment of BMSCs. Conditioned medium collected from SR1078 treated macrophages was used in transwell assay of BMSCs and neutralizing antibodies against CCL3 and IL‐6, BX471 and Tocilizumab, respectively, were also administrated in the transwell system. Crystal violet staining and quantity analysis illustrated that BX471 and Tocilizumab decreased the vertical migration of BMSCs induced by macrophages (Figure 6H,I). Additionally, the results from scratch assay were consistent with the transwell assay (Figure 6J,K). Together, these results demonstrate that RORα promotes migration of BMSCs in a CCL3/IL‐6 dependent manner.

## 3 DISCUSSION

In this study, we outlined the role of RORα in in situ bone healing. Under physiological conditions, significant upregulation of RORα in macrophages was observed in the early stage of bone repair after defects. Macrophage RORα promoted BMSCs recruitment through transcriptional activation of chemokines CCL3 and IL‐6. In diabetes melitus, RORα was not upregulated after bone defect due to deficient upstream IGF1‐AMPK signalling, resulting in impaired bone regeneration. Based on these results, we explored the potential of treating diabetic bone regeneration by targeting RORα and found that the small molecule drug SR1078 can promote diabetic bone regeneration.

Numerous studies have proved the significant role of RORα in regulating physiological activities of tissues and organs. As a constitutive transcription factor, RORα is widely expressed in various tissues such as liver, kidney, skin, and adipose. In addition to its widely known involvement in the functioning of the circadian rhythm system, RORα also plays integral regulatory roles in multiple physiological processes, such as lymphoid tissue development, lipid and glucose metabolism, bone metabolism, and inflammation/immune response.^27^, ^28^, ^29^, ^30^ Staggerer mice, which is a mutant strain with lacked functional RORα, usually die 3–4 weeks post birth due to impeded generation of Purkinje cells,^31^ reflecting the indispensability of RORα in maintenance of homeostasis. In adipose tissue, RORα rhythmically inhibits the thermogenic program of white adipose tissue (WAT).^32^ Lau et al. reported that RORα was a key factor in fat accumulation, staggerer mice had reduced level of serum triglycerides and exhibited resistance to diet‐induced obesity.^33^ Clinical studies also showed that RORα modulated adipose tissue inflammation in obese patients.^34^ In the content of the liver, RORα is an essential regulator in bile acid and cholesterol homeostasis and mediates reprogramming of glucose metabolism in glutamine‐deficient hepatoma cells.^35^, ^36^ In accordance with individual performance, researchers observed abnormal thymus and spleen sizes and impaired cellularity of lymphoid tissue in staggerer mice,^37^ so it is reasonable to assume that RORα is critical in lymphocyte development. Widely expressed in myeloid and lymphoid cells, RORα promotes T and B cell development by providing appropriate microenvironment and controls immune response by regulating cytokines.^19^ The exclusive balance of Th17/Treg cell generation is pivotal for immune homeostasis, RORα was reported to act as an elaborate molecular switch in this teeterboard.^38^ Another study illustrated that RORα regulates the migration and activation of neutrophil, contributing to the host defense against microbial infection.^20^ With the progressive exploration of the biological effects of RORα, its role in bone metabolism is gradually revealed. Meyer et al. demonstrated that RORα is strongly upregulated during the differentiation of BMSCs into osteoblasts. The staggerer mice of deletion within RORα were osteopenic with thin long bones and remarkably decreased total mineral content.^39^ Several in vitro studies have shown that RORα regulated the metabolism of human and mouse osteoblasts and promotes osteogenic differentiation through upregulation of osteogenic mediators such as ALP, OCN, and RUNX2.^40^, ^41^ In the current study, RORα was inhibited in the calvarial tissue of diabetic rats after bone defects (Figure 2). Restoration of RORα function by SR1078 promoted expression of *Col1a1*, *Alp*, *Bmp2*, *Runx2* and *Ocn*, leading to increased bone formation rate (Figure 1). This study illustrates that manipulating RORα to promote bone repair is a viable therapeutic strategy.

Several studies suggested roles of RORα in mesenchymal generation and differentiation. RORα, but not RORβ was expressed in mesenchymal stem cells derived from bone marrow and RORα acts in bone biology by direct modulation of bone matrix component.^39^ Similarly, in human mesenchymal stem cells, RORα was reported to act as a regulatory molecule essential for osteogenic differentiation, genetic intervention of RORα down‐regulated expression of bone sialoprotein and dentin matrix protein 1 and led to failed bone matrix formation and mineralization.^42^ Cho et al. studied RORα in cardiac function and found that RORα was vital in mesenchymal stem cells‐mediated tissue repair.^43^ RORα is increased by IL‐1β and binds to angiopoietin‐like 4, blunting the conversion of macrophages to the proinflammatory phenotype, ultimately facilitating regeneration under pathological conditions. Interestingly, in our study we found that RORα expressed in macrophages promotes recruitment of BMSCs (Figure 5). Taken together, these findings suggest that RORα may be a key node in the crosstalk among different cells and directly or indirectly modulate the tissue regeneration microenvironment.

The molecular mechanism by which RORα exerts its biological effects has been explored in various models. RORα is able to response to extracellular sources of stimulus or endogenous signal mediators and conducts modulation on immune function, mainly through the most characterized pathways including NF‐κB, AMPK and IL‐6/STAT3.^44^, ^45^, ^46^ The regulation role of RORα on LPS response has been intensively studied. Staggerer mice showed elevated levels of IL‐1β, IL‐6 and MIP‐2 in alveolar lavage fluid and were more sensitive to LPS induced lethality.^47^ In another LPS‐induced septic shock model, mice exhibited reduced susceptibility in the absence of RORα,^48^ which was due to passivated macrophages. Treatment with selective RORα inhibitor also reduced the severity of LPS‐induced endotoxemia. These seemingly contradictory results demonstrate the indispensability of RORα in sensing inflammatory stimuli and regulating immune cell function. Specialized pro‐resolving mediators (SPMs) are essential for inflammation resolution, host defene, and tissue regeneration.^49^ RORα was reported to recognize maresin‐1, a classical SPM, activates monocyte phagocytosis and forms a positive feedback loop to promote maresin‐1expression thereby consolidating its anti‐inflammatory effect.^50^ These investigations indicate that RORα can not only sense inflammatory stimuli in the early stage and activate immune response, but also promote resolution of inflammation in the late stage. Melatonin is widely distributed in the organism and has multiple effects such as rhythm regulation and anti‐oxidative stress which are mediated mainly by interacting with specific receptors. Although it is still controversial whether it binds directly to melatonin, RORα is a recognized melatonin receptor and induces the biological function of melatonin.^51^ Choi et al. revealed the link between cholesterol metabolism and osteoarthritis by RORα. RORα in chondrocytes responded to locally elevated cholesterol by upregulating matrix degradation factors MMPs and downregulating anabolic factor SOX9, promoting bone abnormalities.^52^ In the current study, we demonstrated that RORα in macrophages receives upstream IGF1‐AMPK signalling (Figure 4) and transfers the signal to BMSCs by manipulating CCL3/IL‐6 secretion (Figures 5 and 6), ultimately promoting bone regeneration after defect. Under DM conditions, insufficient IGF1‐AMPK signalling impairs the function of RORα. Corroboratively, a recent research found that high glucose deactivates AMPK signalling by production of ROS^53^ and this is consistent with our findings. Our study, along with those existing investigations, suggests that RORα is a key signalling switch that senses microenvironmental cues and drives downstream pathways to modulate cell behaviours.

RORα is a deeply shared molecule in a number of interlinked diseases, thus exploration of therapeutic strategies targeting RORα has significant potential for clinical use. Nowadays, small molecule drugs are the mainstream direction of drug development. Among the new drugs approved by FDA in 2021, small molecules account for more than half of the drugs. RORα is extremely sensitive to small molecule drugs and has potential as a drug target for the treatment of different diseases. In this study, a selective agonist of RORα, SR1078, was systematically administrated to diabetic rats and we did not observe unexpected abnormality in animals, indicating predictable biosafety of the drug. By examing the transcription level of well‐recognized downstream genes of RORα in calvarial tissue, we verified the efficiency of SR1078 (Figure 1). Modulation on RORα‐targeted genes sustained even 8 hr after a single injection, suggesting consistent long‐term effect of SR1078. Finally, through molecular biology, histology and morphology test, we confirmed that SR1078 promotes diabetic bone repair. Overall, we made a preliminary attempt to boost bone regeneration by targeting RORα, further studies of pharmacodynamics and pharmacokinetics are needed to develop a refined application strategy and broaden the scope of clinical applications.

## 4 MATERIALS AND METHODS

Materials and methods are provided in supplementary materials.

## AUTHOR CONTRIBUTIONS

Yufeng Shen, Qingming Tang and Jiajia Wang designed the experiments, analysed the data and wrote the manuscript. Zheng Zhou, Ying Yin and Yifan Zhang made suggestions to the writing of the manuscript and revisions to figures. Wenhao Zheng, Xinyuan Wang, Guangjin Chen and Jiwei Sun participated in conceptualization and methodology. Lili Chen and Qingming Tang supervised the work and critically revised the manuscript. Lili Chen acquired the fundings. All authors contributed to the article and approved the submitted version.

## FUNDING INFORMATION

This research was supported by the National Natural Science Foundation of China for Key Program Projects (82030070) and General Program (82270950), Hubei Provincial Natural Science Fund for Creative Research Groups (2020CFA014), Young Talents Project by the Health Commission of Hubei Province (WJ2021Q059) and the Youth Clinical Research Tund of Chinese Stomatological Association (CSA‐O2020‐10).

## CONFLICT OF INTEREST STATEMENT

The authors claim no competing interest.

## Supporting information


**Data S1:** Supporting Information.

## Data Availability

The data that support the findings of this study are available on request from the corresponding author.
