# Correction to “Targeting RORα in Macrophages to Boost Diabetic Bone Regeneration”

**DOI:** 10.1111/cpr.70084

**Published:** 2025-06-20

Y. Shen, Q. Tang, J. Wang, et al., “Targeting RORα in Macrophages to Boost Diabetic Bone Regeneration,” *Cell Proliferation* 56 (2023): e13474.

Figure 6H was published with incorrect images for the Vehicle and BX471 groups. The correction does not alter any findings and conclusions reported in this article.

The published (incorrect) Figure 6H is provided below:

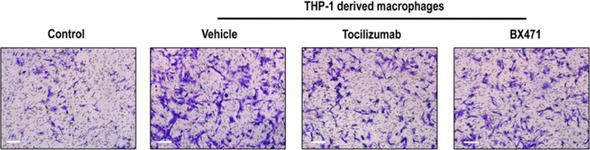

The corrected Figure 6H is provided below:

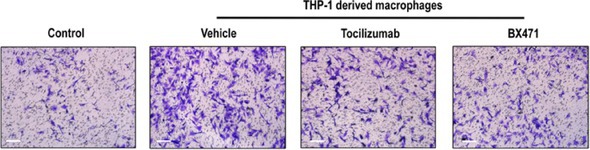

We apologise for this error.

