# Supplementary material for: Targeting RORα in macrophages to boost diabetic bone regeneration
Source: Cell Prolif. 2023 Apr 13;56(10):e13474. doi: 10.1111/cpr.13474 (PMC10542986; doi:10.1111/cpr.13474)
Supplement: Supplementary file 1 — Data S1: Supporting Information. [file CPR-56-e13474-s001.docx]

**Supplementary Materials**

**Materials and Methods**

**Animals**

Male Sprague-Dawley (SD) rats (7 weeks old) were purchased from Beijing Vital River Laboratory Animal Technology Company (Beijing, China) and maintained in the animal laboratory of Tongji Medical College (Wuhan, China). All rats were fed with unrestricted diet and water at a 12 h:12 h light: dark cycle. In this study, all the animal operations involved were permitted by the Institutional Animal Care and Use Committee of Tongji Medical College and were performed in accordance with established guidelines (IACUC number: 3058).

**Induction of type 2 diabetes mellitus in rats**

High-fat diet (HFD) combined with low-dose streptozotocin (STZ) were provided to establish a model of type 2 diabetes mellitus. In brief, SD rats were fed with HFD containing 45% fat, 35% carbohydrates, and 20% protein (D12451, Jiangsu Synergy Biology Co., Ltd) for 4 weeks after adaptation, then STZ (S0130, Sigma-Aldrich) were given by intraperitoneal injection at a dose of 30mg/kg（dissolved in citrate buffer, pH 4.5）. Blood glucose levels were measured using a glucometer (OneTouch Ultra, Lifescan) one week after last STZ injection, and rats with fasting blood glucose levels greater than 11.1 mmol/l were deemed diabetic for next operation.

**Calvarial defect model establishment**

To detect changes in the tissue during bone regeneration, a 4-mm calvarial defect model was established according to previous research^1^. Briefly, rats were gas anesthetized with 2-3% isoflurane. After skin preparation and disinfection, the skin was incised longitudinally along the top of the rat skull until the surgical area was exposed. Subsequently, a 4-mm diameter round cranium was gently peeled from the underlying periosteum with a dental amputation drill, and a digging spoon was used to dig along the edge of the drill to preserve the integrity of the periosteum. The wound area was sutured in layers and the rats were fasting for 4h after surgery.

**Administration of SR1078/ SR3335/IGF1**

DM rats were intraperitoneal injected of SR1078 (10 mg/kg, qd, HY-14422, MCE) or vehicle two days prior to cranial defect modeling and the injection lasted for 9 days. Normal SD rats were intraperitoneal injected of SR3335 (15 mg/kg, bid, HY-14413, MCE) or vehicle two days before the molding and the injection stopped on the seventh day. These two compounds exert effect by regulating the transcriptional activation function of RORα. Therefore, we assayed *Bmal1* and *Clock* expression of endogenous RORα target genes to evaluate the effect of the drug on RORα functional activity. RNA was extracted from the calvaria tissue at 0, 2 and 8 h post the first injection to evaluate the efficiency of the drugs.

A total volume of 30 μl of GelMA hydrogel (10%, EFL-GM-60, EFL) with or without IGF1 protein (2ug, HY-P7203, MCE) was injected in the calvaria defect per animal, and  light-cured for 30 seconds. The incision was sutured in layers.

**Micro-computed tomography analysis**

Rats in each group were sacrificed for tissue assays at 3 d, 7 d,14 d and 28 d after the skull defect model was induced, which received sham operation were taken as the control group. The skull specimens were fixed in 4% paraformaldehyde for 48 h and immersed in 75% absolute ethyl alcohol for micro-computed tomography (micro-CT) scanning (SkyScan 1176, Bruker, Germany) with an accuracy of 9 μm. After reconstructing the original image datasets by NRecon (Bruker, Belgium), DataViewer (Bruker, Belgium) and CTAn (Bruker, Belgium) were utilized to calculate the images of regenerating bony tissue by bone volume fraction (BV/TV) and trabecular thickness (Tb. Th). Each result was measured three times including horizontal and coronal directions.

**Histological analysis**

After micro-CT scanning, the calvaria were decalcified, paraffin-embedded, and sectioned (5). Masson's trichrome staining was performed using a commercial Masson Staining Kit (S191006, Pinofi) to observe the degree of collagen maturation.

For iImmunohistochemistry (IHC) analysis, after deparaffinized and rehydrated, the sections were placed in citric acid antigen repair buffer (pH 6.0) and heated in a microwave for antigen retrieval. The blocked sections were incubated with the primary antibody of Collagen I (1:150, A1352, Abclonal) , ALP (1:1000, A0514, Abclonal), RORα (1:400, ab256799, Abcam) and RUNX2 (1:1000, ab192256,Abcam). Then horseradish peroxidase (HRP)-coupled secondary antibodies were used to detect the corresponding primary antibodies. Finally, the samples were visualized by the chromogenic substrate diaminobenzidine (DAB) substrate. The images were captured using a slide scanner and quantity analyses were conducted using the Image J software.

For immunofluorescent (IF) analysis, after antigen retrieval，the sections were blocked in 3% BSA. Alexa Fluor 488 and Cy3-conjugated secondary antibody (1:200, SA00013-2, Proteintech) and 4′, 6-diamidino-2-phenylindole (DAPI, 1:1000, Beyotime) were applied for incubation for visualization following treatment with the primary antibody RORα (1:100, ab278099, Abcam), as well as CD68 (1:200, sc20060, Santa Cruz).

**Enzyme Linked Immunosorbent Assay (ELISA)**

A Rat IGF-1 enzyme-linked immunosorbent assay (ELISA) kit (HYCEZMBIO, HY30977) was used to measure serum IGF1 protein levels in normal and DM rats. 50 uL of standard and serum samples were added to the wells, followed by 100 uL of HRP-conjugated antibody for 1 h according to the manufacturer’s instructions. After washing, 50 µL substrate A and 50 µL substrate B were added separately to incubate for 15 min and then the reaction was stopped with stop solution. The OD values were measured at 450 nm.

**Cell culture and stimulations**

**Bone marrow derived macrophages(BMDMs).** Male rats of SPF Grade (180-200g) were executed by cervical dislocation, soaked in alcohol and sterilize for 5min before separation. After removing the skin and muscle tissue on the surface of femur, the bone marrow was gently washed out using a sterile syringe. The obtained suspension was mixed with red blood cell lysate to remove red cells and incubated in low-sugar DMEM supplemented with 10% FBS,20% L929 supernatant, 100 U/mL penicillin and 100 g/mL streptomycin (1% p/s).

**Bone marrow mesenchymal stem cells（BMSCs）.** BMSCs were isolated from 60-80g rats using a whole bone adherent method as described previously. The cells were grown in alpha modification of Eagle’s medium (α-MEM) containing 10% (v/v) fetal calf serum (Gibco) and 1% p/s.

**THP-1.** THP-1 cells（ATCC, American）were cultured in RPMI supplemented with 10% FBS and 1% p/s. After the cells grown to an appropriate density, phorbol 12-myristate 13-acetate (PMA, 100 ng/ml, Selleck) was administrated for 48h to induce cell differentiation, followed by 1 day in RPMI without PMA. THP-1 cells were choosed for ChIP assay and signaling pathway-related studies for they have high proliferative capacity and can be stably induced into macrophages.

THP-1 derived macrophages were treated with IGF1 (100 ng/ml, HY-P70788, MCE) or IGF1R inhibitor Picropodophyllin (PPP, 5μM, HY-15494, MCE) under 25mM high-glucose microenvironment for 12, 24 h to examine *RORA* mRNA levels, and pretreated with IGF1 for 1 h followed by administration of AMPK/MAPK activator or inhibitor (MCE) for 23 h to detect the relative protein levels by Western Blot.

**Lentivirus infection**

Cells were infected with lentivirus gene expression vector (GV492) containing *Rora* (XM_008776764) gene sequence for *Rorα* overexpression. Element sequence: Ubi-MCS-3FLAG-CBh-gcGFP-IRES-puromycin. The CRISPR-Cas9 system with CV279 vector (element sequence: MCS-EF1a-Cas9-FLAG-P2A-puro) was used to knockdown the expression of *Rorα* in BMDMs. Lentivirus was obtained from Genechem Co., Ltd. (Shanghai, China). BMDMs seeded in 6-well plates at 1×10^6^ cells/well were incubated with the viral supernatants (MOI=20) in complete medium, and the medium was replaced with standard culture media 48 hr post infection. Transfection efficiency was verified by qRT-PCR after 4 μg of puromycin/ml selection.

**Cell migration assay**

Cell migration ability was measured by transwell assay and scratch experiment. For the transwell assay, 1×10^4^/mL BMSCs were seeded in the upper chamber (8-μm pore; Corning Life Sciences) in 200ul α-MEM (1 % FBS) and co-cultured with conditioned medium from BMDMs. For neutralization test，CCL3-neutralizing antibodies BX471（1 μM, HY-12080A, MCE）and IL6-neutralizing antibodies Tocilizumab（1 μM, HY-P9917, MCE）were added to the co-culture system separately. After 24 h, 4% PFA and 1% crystal violet were used to fix and stain the cells that migrated to the underside of the membrane. The migrated cells were quantified under a light microscopy (Nikon Eclipse TE2000-S, Japan).

For scratch assay, BMSCs were cultured to create a confluent monolayer in a scratch-specific six-well plate. The removable scratch plate was moved out after 24 h and the conditioned medium was added to continue incubation for 24 h. Migrated cells were then tracked using a light microscopy to the respective cell migration rate was quantitated.

**Cell viability assay**

Conditioned medium was obtained from supernatant of RORα overexpressed or knockdowned BMDMs. EdU incorporation assay and CCK8 assay were performed to test the vitality of BMSCs cultured in conditioned medium. EdU staining was performed using the BeyoClick™ EdU Cell Proliferation Kit with Alexa Fluor 488 (C0071S, Beyotime) according to the manufacturer’s instructions, cells were incubated with EdU for 4 h. For CCK8 assay, 10 ul CCK8(C0038, Beyotime) was added to each well and the plates were incubated for 2 h at 37°C.The optical density (OD) at 450 nm was recorded.

**Western blotting (WB)**

Cells were harvested and lysed in prechilled RIPA buffer containing a protein phosphatase inhibitor cocktail (P1260, Solarbio). Extracted total proteins (30µg) were diluted with 1×loading buffer and boiled at 95°C for approximately 10 min. The proteins were separated by Gradient SDS-PAGE (10%) and transferred onto PVDF membranes. After blocked with 5% nonfat milk, the membranes were incubated with primary antibodies at 4℃ overnight and incubated with corresponsive secondary antibodies. The primary antibodies used were as follows: RORα (1:1000, ab278099, Abcam); MEK1(1:2000, 2352s, CST); p-MEK1(1:1000, AP1021, Abclonal); AMPKα1 (1:1000, ab32047, Abcam); p-AMPKα1 (1:1000, AP1002, Abclonal); β-Actin (1:1000, AC006, Abclonal). The images were captured using Chemiluminescent imaging system (ChemiScope S6 SE, China) and quantitated in Image J Software.

**Quantitative reverse transcription-polymerase chain reaction (qRT-PCR)**

Total RNA was extracted and purified from cranial bone tissue samples according to the instructions of the Rapid Bone Tissue RNA Extraction Kit (RN5401, Aidlab). TRIzol method was mainly used to extract RNA in cellular experiments. Complementary DNA (cDNA) was acquired by reverse transcription of the high purity RNA via the HiScript III RT SuperMix (R323-01, Vazyme), and then SYBR Green detection reagent (Q711-02, Vazyme) was applied for qRT-PCR analysis. The relative expression levels were calculated using the 2^−ΔΔCt^ method. *Gapdh* expression was used for normalization of results. Table 1 shows the primer sequences.

**Immunocytochemistry**

THP-1 cells were seeded at a density of 1×10^5^ cells in 24-well culture plates plated with cell climbing slices, then pharmacological intervention was performed by adding activators or inhibitors of AMPK under 25Mm high-glucose condition after PMA induction. Cells were fixed and stained with antibodies of RORα (1:100, ab278099, Abcam). Images were obtained by a laser scanning confocal microscope.

**Chromatin immunoprecipitation (ChIP) assay**

ChIP assays were performed using Chromatin Immunoprecipitation Kit (P2078, Beyotime) according to the procedures provided by the manufacturer. Chromatin solutions were precipitated using normal rabbit IgG and anti-RORα (1ug, sc-518081x, Santa Cruz) at 4 ℃ overnight. Precipitates were detected by qPCR. The primer sequences are shown in Table 2.

**Statistical analysis**

All data were presented as mean and standard deviation and the results were applied Shapiro-Wilk test for normal distribution and Brown-Forsythe test for homogeneity of variance before one-way or two-way analysis of variance (ANOVA). All statistical analyses were performed using GraphPad Prism software version 9.1.0 (GraphPad Inc.). *P* < 0.05 was set as the threshold to indicate significant difference between groups.

**Table 1 The primers used for qRT-PCR**

| Primer | Forward (5′- 3′) | Reverse(3′- 5′) |
| --- | --- | --- |
| *r-Gapdh* | AGGGCTGCCTTCTCTTGTGAC | ATCTCGCTCCTGGAAGATGGTG |
| *r-RORα* | TCCAGCAGATAACGTGGCAG | CACCTCTAGAGAGCCTGCTTTT |
| *r-Bmal1* | GACTTCGCCTCCACCTGTTCAA | TCATTGTCTGGTTCACTGTCTTCGT |
| *r-Clock* | CCAACTCCTTCTGCCTCCTCCA | ACCTCCGCTGTGTCATCTTCTCA |
| *r-Osx* | GCTGCCTACTTACCCGTCT | CCCACTATTGCCAACTGC |
| *r-Alp* | GACAAGAAGCCCTTCACAGC | ACTGGGCCTGGTAGTTGTTG |
| *r-Bmp2* | TGGGTTTGTGGTGGAAGTGGC | TGGATGTCCTTTACCGTCGTG |
| *r-Runx2* | CCGATGGGACCGTGGTT | CAGCAGAGGCATTTCGTAGCT |
| *r-Ocn* | GGTGCAAAGCCCAGCGACTCT | GGAAGCCAATGTGGTCCGCTA |
| *r-Il6* | GACAAGAAGCCCTTCACAGC | ACTGGGCCTGGTAGTTGTTG |
| *r-Ccl3* | GCTGCTTCTCCTATGGACGG | TCTCTTGGTCAGGAAAATGACAC |
| *r-IGF1* | CCCGGGACGTACCAAAATGA | AGCCTGTGGGCTTGTTGAAG |
| *h-GAPGH* | GCACAAGAGGAAGAGAGAGACC | AGGGGAGATTCAGTGTGGTG |
| *h-RORA* | AAACATGGAGTCAGCTCCG | CATACAAGCTGTCTCTCTGC |
| *h-IGF1* | GCTCTTCAGTTCGTGTGTGGA | GCCTCCTTAGATCACAGCTCC |
| *h-CCL3* | TGCACTTTATGACGCACTCAC | TGTCCAAAAACACGAAATCATGC |
| *h-IL6* | GCTCTCTGCAACCAGTTCTCT | TCGCTTGGTTAGGAAGATGACA |

**Table 2 ChIP assay primers**

| Primer |  | Forward (5′- 3′) | Reverse(3′- 5′) |
| --- | --- | --- | --- |
| *CCL3* | #1 | TAGAGACCCTTCCACACCACTG | CACCCAGGGACCTATCACACAAA |
|  | #2 | TTCAGTTCTTTGCCTCTGGGA | CACTCACAGGAGAAACCATTTCC |
| *IL-6* |  | CGCCTGTAAACCCAGCACTT | TGGCATGATCTTGGCTCACT |

**Reference:**

1. Qi, C., et al., *A sericin/ graphene oxide composite scaffold as a biomimetic extracellular matrix for structural and functional repair of calvarial bone.* Theranostics, 2020. **10**(2): p. 741-756.
